# Innate Synchronous Oscillations in Freely-Organized Small Neuronal Circuits

**DOI:** 10.1371/journal.pone.0014443

**Published:** 2010-12-28

**Authors:** Mark Shein Idelson, Eshel Ben-Jacob, Yael Hanein

**Affiliations:** 1 School of Electrical Engineering, Tel-Aviv University, Tel-Aviv, Israel; 2 School of Physics, Tel-Aviv University, Tel-Aviv, Israel; Indiana University, United States of America

## Abstract

**Background:**

Information processing in neuronal networks relies on the network's ability to generate temporal patterns of action potentials. Although the nature of neuronal network activity has been intensively investigated in the past several decades at the individual neuron level, the underlying principles of the collective network activity, such as the synchronization and coordination between neurons, are largely unknown. Here we focus on isolated neuronal clusters in culture and address the following simple, yet fundamental questions: What is the minimal number of cells needed to exhibit collective dynamics? What are the internal temporal characteristics of such dynamics and how do the temporal features of network activity alternate upon crossover from minimal networks to large networks?

**Methodology/Principal Findings:**

We used network engineering techniques to induce self-organization of cultured networks into neuronal clusters of different sizes. We found that small clusters made of as few as 40 cells already exhibit spontaneous collective events characterized by innate synchronous network oscillations in the range of 25 to 100 Hz. The oscillation frequency of each network appeared to be independent of cluster size. The duration and rate of the network events scale with cluster size but converge to that of large uniform networks. Finally, the investigation of two coupled clusters revealed clear activity propagation with master/slave asymmetry.

**Conclusions/Significance:**

The nature of the activity patterns observed in small networks, namely the consistent emergence of similar activity across networks of different size and morphology, suggests that neuronal clusters self-regulate their activity to sustain network bursts with internal oscillatory features. We therefore suggest that clusters of as few as tens of cells can serve as a minimal but sufficient functional network, capable of sustaining oscillatory activity. Interestingly, the frequencies of these oscillations are similar those observed *in vivo*.

## Introduction

Intense synchronous firing activity is one of the hallmarks of developing and active neural networks. These synchronized events have been extensively mapped both in in vivo and in vitro investigations [1]. It was shown that the collective activity of neuronal networks is marked by bursts of intense firing (several hundreds of milliseconds), separated by longer periods (several seconds) of only sporadic firing [1], [2]. Synchronous activity patterns are thought to play a major role in the development of the neuronal circuitry [3], as well as in information processing (coding), in sustaining memory and in regulating network level activity [1], [4], [5], [6], [7], [8]. More specifically, it was shown that tetanic electrical stimulations induce network-level pathway-dependant modifications to coupling strength [4], [9]. The collective activity of neurons was shown to have both complex temporal organization, as well as auto-correlations over long time periods [5], [6]. In slices and in vivo this spontaneous activity involves network level oscillations which are characterized by high coherency over distant network sites [10]. In addition, repetitive spatio-temporal patterns of firing with defined propagation schemes were identified in the network spontaneous activity [11], [12], [13], [14]. These patterns could be artificially evoked by targeted electrical [15] and chemical [16] stimulations.

Despite the ubiquitous nature of synchronized activity patterns in neural networks, and the growing understanding of neuronal function, the manner by which a network of neurons and glia cells can give rise to synchronized activity is still under intensive research [11], [14], [17], [18], [19]. Understanding the functional properties of neurons has evolved from a basic view of threshold-dependent pulse generators that perform simple activity integration to highly complex processors that perform a variety of self regulated computational tasks [20]. Interestingly, this evolved view of single neurons is insufficient to describe the collective dynamics and activity patterns of connected neurons [17].

Understanding how the electrical activity properties vary upon crossover from single neurons to the network level, may provide the insight needed to reveal the innate properties of neuronal network dynamics. This understanding is particularly intriguing as it may be implemented in various fields, ranging from neural network modeling [21], [22], network theory [23], and engineering and bio inspired (bionic) devices [16], [24], to name just a few.

To characterize the transition from single cells to neuronal populations in terms of their electrical activity, we engineered small isolated neuro-glia clustered networks of various sizes and examined their collective activity. Clusters are of particular interest as they form spontaneously in vitro with only minimal external intervention [25], [26], [27], [28], [29]. Moreover, clustering characterizes many biological brain networks. We promote the formation of clusters by exploiting the tendency of dissociated neuronal cells to self-organize into patterned architectures due to their preferential attachment to cell-attracting chemistries, such as poly-d-lysine (PDL) or to rough surfaces, such as carbon nanotubes (CNTs) [26], [28]. By controlling their dimensions, we can systematically form and map the activity of neural networks with well identified cell numbers, ranging between several cells up to several hundreds. As we show below, such small systems demonstrate well characterized activity reflecting a clear transition from sporadic to well synchronized network level activity.

## Results

### Engineering freely organized small neuronal circuits

**Figure 1:**
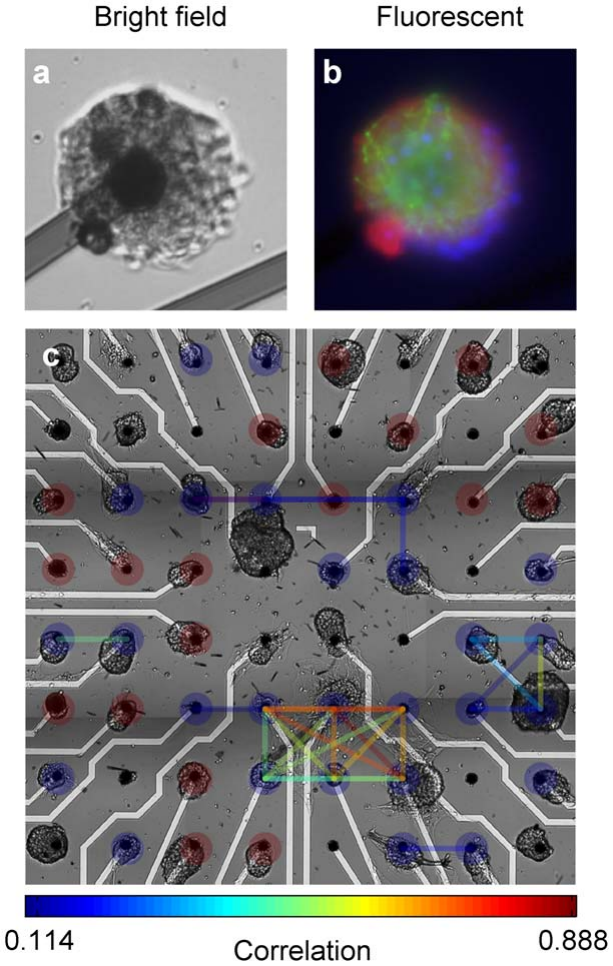
Isolated cluster formation on CNT multi electrode array (MEA). (a) A bright field image of a neuronal cluster on a CNT electrode. The electrode diameter is 30 µm and the inter electrode distance is 200 µm (b) A fluorescent microscope image of the cluster in (a), stained for cell nuclei (DAPI-blue), glia (GFAP-green) and neurons (TUJ1-red). (c) A bright field image of clusters on a MEA chip. Color coded lines show the Pearson correlation between the electrical activities of all cluster pairs above a threshold of 0.1. The electrically isolated clusters (red full circles) were distinguished from linked clusters (blue full circles) both functionally (no significant correlations to other clusters) and visually (no apparent extensions to other clusters).

Isolated small neuronal circuits or neuronal clusters made of a few to several hundreds of neurons and glia cells were engineered using rectangular arrays of adhesive micro islands made of CNTs or PDL deposited on planar recording electrodes [28]. Dissociated neurons and glia cells placed on such integrated multi electrode arrays or neuro-chips, self-organized into small isolated clusters with dimension between 20–120 µm in diameter (Figure 1). The activity of each cluster was recorded by an underlying micro-electrode allowing recordings from as many as 60 isolated islands in parallel.

To determine the cluster size and cellular arrangement within each cluster, neurons, glia and cell nuclei were specifically labeled (with GFAP, TUJ1 and DAPI, respectively) and imaged (Figure 1b). We found that each cluster was made of both neurons and glia cells spatially arranged as glia carpets with overlying neurons (see details in Text S1). Moreover, neurons in all clusters exhibited extensive neurite growth (Figure 1b) and synapse formation (Figure S1). The area of each cluster was used as a measure of its size and the number of cells in each cluster was estimated from the cluster size (see details in Text S2 and Figure S2).

### Emergence of network events in small neuronal circuits

**Figure 2:**
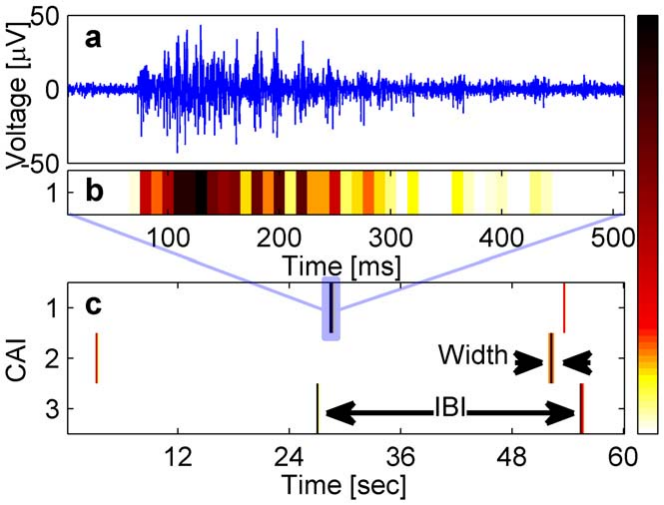
Network bursts in small isolated clusters. (a) A typical voltage trace of a network burst recorded in a single electrode. (b) The cluster activity intensity (CAI) (calculated in 10 ms bins) of data shown in (a). (c) Raster plot of the activity intensity of three isolated clusters recorded from the same MEA chip (the blue rectangle marks the time window in (b)). Recorded activity is characterized by network bursts (NBs).

After two weeks in vitro, neuronal clusters exhibited noticeable spontaneous activity (Figure 2a). We note that since each cluster was recorded by a single electrode, the recorded activity represents the sum of firing of many neurons. Consequently, we defined a population-level cluster activity intensity measure (CAI)(Figure 2b) which is estimated from the recorded voltage waveform (see details in Text S3, Figure S3 and Figure S4). The recorded spontaneous activity of isolated clusters was marked by synchronized bursting events or network bursts (NB). These network events are similar to the network bursts observed in large homogeneous networks composed of hundreds of thousands of cells. More specifically, they are characterized by short time windows of intense neuron firing followed by longer intervals of sporadic firing (Figure 2c). Additional similarity is in the intra burst patterns and in the burst statistics. These topics are presented in the next sections.

The activity of all the clusters in each neuro-chip was recorded and analyzed. During the self-organization process, some of the clusters became linked by bundles of axons. To distinguish between isolated and linked clusters, we calculated the Pearson correlation between the activities of all cluster pairs. As can be seen in Figure 1c, the activity of some clusters is correlated, but there are some pairs of clusters with vanishingly small correlations. The latter are the isolated clusters whose activity we analyzed while connected clusters, which had significant correlation with at least one other cluster, were eliminated from the analysis. The identification of linked clusters was also validated by visual inspection (bright field and fluorescent images). Finally, the temporal loci, as well as the width for each NB were detected (see details in Text S4 and Figure S5) and the inter burst intervals (IBIs) were calculated.

### Temporal characteristics of network bursting

**Figure 3:**
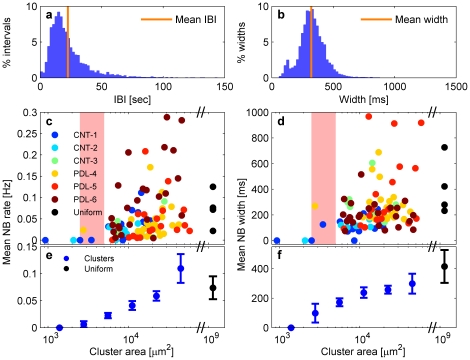
Collective synchronized activation in small clusters. (a) and (b) show the IBI and the NB widths distributions, respectively, in a typical isolated cluster. The IBI distribution is characterized by an asymmetric distribution with non vanishing probability for the occurrences of long intervals, while the width distribution is characterized by a typical time scale. The average NB rate (1/IBI) and width (orange line in (a) and (b), respectively) are calculated for every cluster separately and are presented as a function of the cluster's size (in log scale) in (c) and (d), respectively. Ninety-seven clusters from six different cultures (three CNT and three PDL-coated electrodes) were analyzed. Four additional cultures of uniform networks were analyzed for comparison (black dots). The average NB rate and width were calculated for consecutive and equal time bins on a logarithmic scale in (e) and (f), respectively. The error bars represent the standard deviation of the average value in every bin. The high variability in the activity properties observed in clusters is also observed in uniform networks of 10^6^ cells (black dots). The red area (corresponding to clusters of 2500–5000 µm^2^) marks the estimated transition to spontaneously bursting clusters (its upper limit corresponds to approximately 40 cells).

It was previously shown that inter-burst-intervals (IBIs) of NBs are characterized by a long tail multi time-scale distribution [5]. Interestingly, the IBI distribution of our small clusters revealed similar behavior (Figure 3a). The general trend is that smaller networks exhibit network bursting with lower rates (IBIs on the order of minutes), while larger clusters exhibit faster network bursting (IBIs on the order of seconds). The fast bursting rate is comparable to that of large homogeneous networks (Figure 3c). Despite this apparent trend, there is also significant variability in the network bursting rate between different clusters of the same size. Additionally, in some of the clusters, the IBI distribution was characterized by more than one peak due to the fact that many NBs were grouped into bursts of NBs with short intervals (on the order of one second) between them. In Figure 3b we show the NB width distribution for a typical cluster. This distribution has a narrow typical time scale, as shown in Figure 3b, with high variability in the mean NB width between different clusters (Figure 3d).

### Closer inspection of the effect of the cluster size

The effect of the size of the clusters on the network bursting rate and event width is shown in Figures 3c–f. Figures 3c,d show results from all analyzed clusters while figures 3e,f show the average rate and width, respectively, calculated in consecutive logarithmic time windows (error bars represent the standard deviation of the mean value – standard deviation of the data points in each bin divided by the square root of the number of data points). The most intriguing feature is the onset of network activity at clusters with as few as several tens of cells. Apparently, clusters larger than 5000 µm^2^, corresponding to about 40 cells (see details in Text S2), already exhibit synchronized network activity. We therefore approximate that the upper limit for the onset of synchrony is about 40 cells. Based on our data we can estimate that a transition occurs within a cluster area range of 2500–5000 µm^2^, corresponding to about 20–40 cells (marked by the red box in Figures 3c,d). None of the smaller clusters in our experiments (below 2500 µm2) exhibited synchronized network activity. This result suggests the existence of a minimal network size which is required to generate and sustain collective activity. It is important to note that such small clusters (assigned zero NB rate in Figure 3c) do exhibit tonic single spike activity. However, the single neuron firing is not sufficient to generate collective network bursts. There are also silent small clusters that did not exhibit any electrical activity. Those were eliminated from the analysis (see Methods for further details). As was mentioned above, the NB rate and the NB width appear to increase with cluster size (Figure 3c,d – colored dots). This increase converged to the NB rate and width of large uniform networks of 10^6^ cells (Figure 3c,d - black dots).

### Synchronous oscillations within the network bursts

We now inspect the internal temporal features of the network events. First, similarly to large homogeneous networks [19], most of the network events recorded in isolated clusters have a stereotypical temporal profile with a fast rise (tens of milliseconds) in the activity intensity, followed by a slower activity decay (hundreds of milliseconds), as is shown in Figure 2a. This activity profile reflects the fact that many neurons are rapidly activated at the onset of NBs and are gradually relaxed or inhibited with time leading to the NB intensity decay.

**Figure 4:**
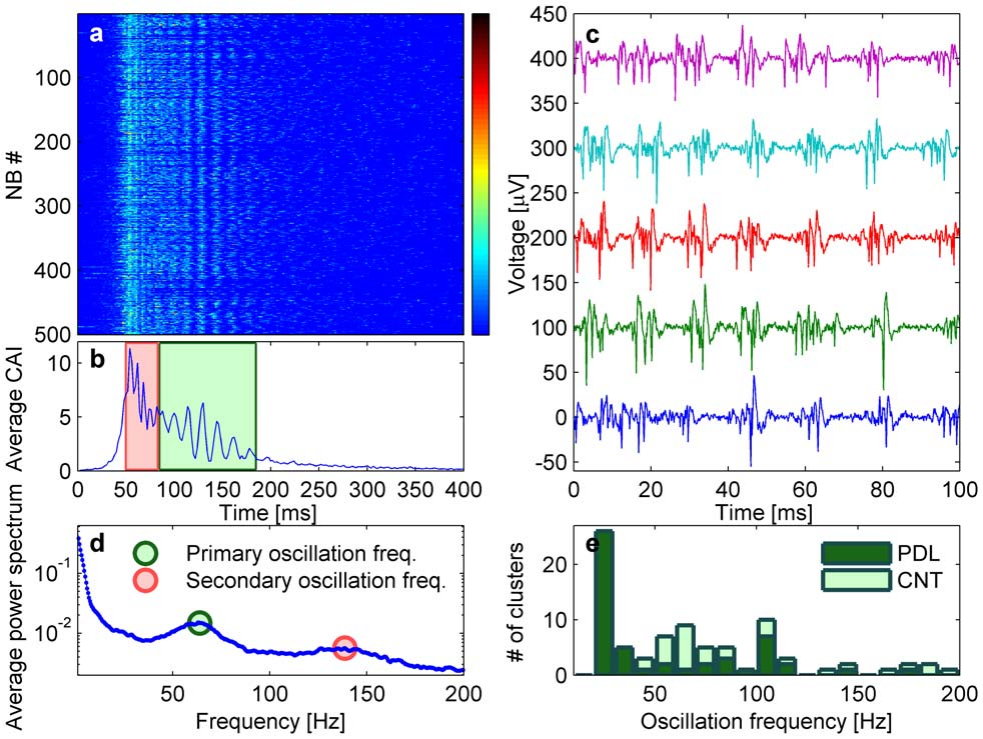
Spontaneous oscillations in small isolated clusters. (a) Five hundred consecutive CAI traces (400 ms long) from one cluster temporally aligned to maximize the correlations between them. (b) The average over the CAI traces in (a). (c) The voltage traces of 5 consecutive NBs from (a) during the time window marked by a green rectangle in (b). The fine details of each voltage trace are different but all show oscillating activity which involves the synchronous firing of several superimposed spikes from several neurons. (d) The average power spectrum of the NBs in (a) presented in log scale. The second and third peaks at 64 Hz and 139 Hz represent the primary and secondary oscillations in the cluster occurring during the time windows marked by the green and red rectangle in (b), respectively. (e) The oscillation frequency histogram, presented separately for clusters grown on CNT and PDL.

The overall similarity between consecutive NBs described above reflects a much more significant correspondence between them. To reveal this correspondence, the CAI traces of consecutive NBs were aligned in a way that maximizes the correlation between them, as shown in Figure 4a. The existence of repeated patterns and synchronous oscillations are readily apparent (Figure 4a,b). Although the temporal motif of synchronous oscillations is conserved for many consecutive network bursting events (Figure 4a shows 500 consecutive events from a single cluster), each event has its own temporal fingerprint. The exact duration of the oscillations varies between consecutive NBs (Figure 4a). In addition, some of the NBs are aborted before the onset of network oscillations (Figure 4a). Such aborted network bursts were previously reported in uniform cultures [19]. Unique finger prints were also detected on the millisecond scale. In Figure 4c we show a series of voltage profiles of consecutive network events from Figure 4a. Evidently, while they all show the same pattern of synchronous oscillations on the global scale (tens of milliseconds), each event has its own temporal profile on the millisecond scale. These observations are consistent with the idea that synchronous oscillations are generated by synchronized firing of several neurons, with different neurons participating in different network events. Alternatively, it is possible that the same neurons participate but with a different phase delay in their firing [30]. The general intra-burst activity patterns described above were consistently observed for the majority of the clusters.

Examining the averaged power spectra (see Methods) of the NBs revealed that they are typically characterized by several well defined peaks (Figure 4d). The low frequency peak with the highest amplitude is associated with the gradual (long time) decay in firing intensity towards the end of the termination of the network event. The second peak (green circle in Figure 4d) is associated with the primary synchronous oscillations during the decay in the firing activity (green rectangle in Figure 4b). In a small fraction of the clusters (12%) an additional peak in higher frequencies was detected. These peaks were classified as secondary oscillations (red rectangle in Figure 4b and red circle in Figure 4d). The distribution of both primary and secondary oscillations across the whole population of recorded clusters is presented for clusters grown on CNT and on PDL in figure 4e. Clusters that did not exhibit NBs or those with a very small number of NBs (<100) were not analyzed. In total, 73% of the clusters exhibited oscillations (71% of the CNT clusters and 74% of the PDL clusters), 20% did not show detectable oscillations and 7% were not analyzed due to their low activity. For most clusters, the frequency peaks appear in the gamma range (25–100 Hz). The average oscillation frequencies of the clusters grown on CNT islands was 92±46 Hz (mean±std) (distribution peak at 65 Hz), and the average oscillation frequencies of clusters grown on PDL islands was 54±38 Hz (distribution peak at 25 Hz). No significant correlation was found between the oscillation frequency and the cluster size (correlation coefficient = −0.15, p-value = 0.19). In addition, the oscillations were abolished following the addition of 30 µM Bicuculline (see details in Text S5 and Figure S6), a γ-Aminobutyric acid (GABA) receptor inhibitor, suggesting that inhibitory neuronal activity is required to sustain the oscillations.

### Mutual events in two coupled clusters

**Figure 5:**
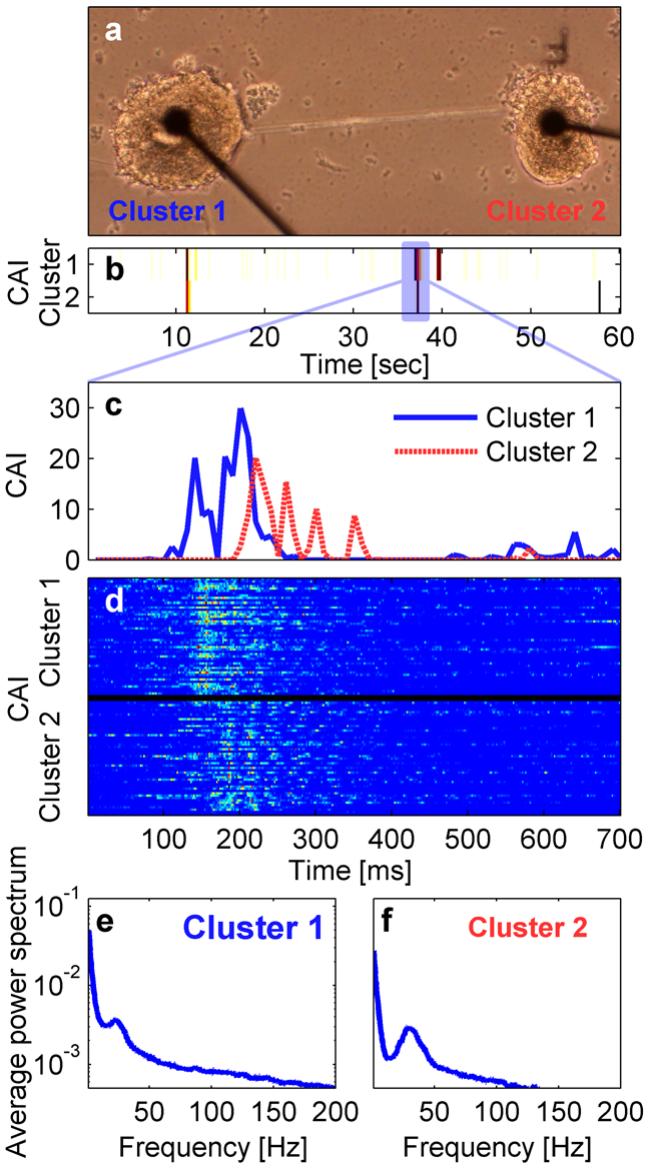
Activity in small coupled networks. (a) A bright field image of an engineered network of two clusters coupled by a bundle of neurites (substrate: PDL, Electrode diameter is 30 µm, distance between electrodes is 500 µm) (b) Activity intensity (250 ms bins) during a 60 sec window of clusters 1 and 2 showing that both exhibit network bursts (color code is the same as in Figure 2c). (c) Activity intensity (10 ms bins) during a 700 ms window in (b)(marked by the transparent blue rectangle). Blue and red lines represent activity of clusters 1 and 2, respectively. (d) Activity intensity (2 ms bins) during 700 ms windows of 50 consecutive NBs (color code is the same as in Figure 4a). The fine details in the activity of both clusters are different for each NB. (e) and (f) show the average power spectrum extracted from the NBs in cluster 1 and 2, respectively, presented in a log scale. Both clusters 1 and 2 exhibit a peak at 23.4 Hz and 28.8 Hz, respectively.

Our unique neuronal engineering scheme also allows us to study the activity of two clusters coupled by a bundle of neurites (Figure 5a). Typical recordings of such a system reveals that a network of two coupled clusters can exhibit mutual NBs (Figure 5b). We note that in addition to the mutual NBs, each cluster may also exhibit individual NBs where a NB is activated in one cluster but does not propagate to the other cluster.

In the case of mutual events, there is a relatively long (up to hundreds of milliseconds) time delay between the onset of synchronized activity in the two clusters (Figure 5c, d). We note that since the clusters are coupled bi-directionally, we cannot determine that the activity during mutual events was initiated individually in one cluster and propagated to the other cluster. A more plausible scenario is that the NB was first initiated in one of the clusters due to mutual interaction between neurons in both clusters, and later appeared with a time delay in the other cluster. For this reason, an adequate description of the activity propagation should be in terms of time delays in a mutually synchronized network, rather than by simple triggering. Similarly to the findings in large coupled networks [31], we found a spontaneous emergence of master/slave asymmetry in the activation delays in both clusters. The activity of one specific cluster preceded the activity of the other cluster in the majority of mutual events (Figure 5d).

During the mutual events, the activity of each cluster exhibits the same temporal features that are observed for the isolated clusters including the existence of synchronous oscillations. We also found that each cluster has its own temporal identity. In the example presented in figures 5e,f we show that each cluster has synchronous oscillations with specific characteristic frequency, 23.4 Hz and 28.8 Hz, for clusters 1 and 2 respectively. We note that these frequencies are within the range of the typical frequencies observed in isolated clusters grown on PDL islands. This implies that the coupling between the two clusters leads to mutual events but does not alter the existence and typical frequency of the synchronous oscillations. As shown for isolated clusters, the oscillations in coupled clusters are abolished by application of 30 µM Bicuculline (see details in Text S5 and Figure S6).

## Discussion

It is often suggested that cooperative activity within neuronal assemblies enhances their information processing capacity compared to that of isolated neurons [32]. However, the transition in activity from the single neuron to the population level has not been mapped experimentally. For example, it is unclear as to what is the minimal number of cells which is required to define a functional network. Here we showed that neuro-glia cortical cell assemblies of approximately 40 cells already exhibit spontaneous network-level bursting activity, which is absent in smaller (below approximately 20 cells) neuronal clusters (Figure 3c). While some of these clusters contained at least one spontaneously active cell, they failed to elicit spontaneous network bursts. This suggests that despite the high range of network sizes within which NBs are maintained, there exists a minimal network size below which the requirements for collective spontaneous activation are unmet. Interestingly, theoretic considerations as well as physiological data suggest the existence of minimal networks of similar size in real brains [32].

We also found that the statistical parameters of the network activity scale with the cell number. Namely, the rate and width of the network bursts were found to increase with cluster size. Interestingly, these parameters converge to that of large networks (∼10^6^ cells). Large networks usually exhibit NB rates of several NBs per minute. These rates are already achieved by clusters of about 100 cells. This suggests that increasing the cell number above 100 cells does not dramatically change the statistical activity properties of the network. It is worth noting that clusters of similar sizes exhibited high variability in the NB rate and width (Figure 3c,d). This may suggest that additional unmeasured cluster properties (beyond cell number) influence the cluster activity. This high variability is consistent with previous reports which concerned large networks [33]. Although previous measurements of small homogeneous networks [5] and model simulations [34] are consistent with our data, this is the first time that the effect of population size was quantitatively studied.

Invariability between entirely different clusters is an additional, important feature of the data described above. It is commonly believed that neuronal circuit architecture determines its functionality. However, it is not clear to what extent does variability in circuit parameters, such as neuronal excitability and synaptic connectivity, relate to variability in the circuit's functionality. Theoretic models [35], as well as physiological data [36], suggest that neuronal circuits are able to maintain similar functionality with variable architectures. The organization of our clustered networks into connected circuits was self-executed by the neurons and the glia cells. Consequently, the exact architecture of each neuronal cluster was different. In addition, our cortical cultures contained many cell types, each having distinct morphological and function features [37]. For small clusters, this implies that the distribution of cell types was different for every cluster. It is also likely that the exact connectivity scheme of the cells within each cluster was different. Despite the above variability, all the clusters showed spontaneous persistent collective activity in the form of NBs with markedly similar features (Figure 3,4). This hints that almost every network, independently of its architecture and size, self regulates its activity to sustain persistent activity patterns. This assumption is supported by the well known existence of both redundant cellular mechanism that support synchronization [38], and homeostatic mechanisms that support activity regulation [7], [18].

We have also demonstrated that our small clustered networks exhibit persistent network-level oscillation in the range of 25–100 Hz (Figure 4e). These frequencies are of particular interest as they are manifested in brain activity and are typically associated with functional properties such as temporal encoding, sensory binding, and storage and recall of information [10], [39]. Oscillations were observed in most of the analyzed clusters, suggesting that they are a generic property of small neuronal populations rather than the outcome of specific network architecture. In addition, the oscillations were more prominent at the decaying phase of the NBs. Such delayed activation may suggest that the oscillatory state is the outcome of a collective dynamics process that has to evolve until oscillations appear. Alternatively, the time delay may be related to a delayed activation of a synchronizing mechanism.

It was previously shown, both in experimental and in theoretical studies, that oscillations in the cortex are generated by a combination of network interactions and cellular mechanisms [39]. More specifically, the combined action of recurrent excitation and modulating inhibition (mediated by GABAergic receptors) are required to produce the oscillations. In addition, gap junctions were shown to play an important role in synchronizing neurons during oscillations [40]. Clearly, investigation of the mechanisms mediating the oscillatory behavior in our small clusters is of major importance. We have shown that the oscillations are inhibited by blocking GABAergic receptors (Figure S6). However, only a detailed examination will determine the relevance of the oscillations to the ones observed in vivo. The uniqueness of our setup enables us to study how activity patterns, in general, and the oscillations, in particular, are modulated by different network configurations, i.e., by the ratio between excitatory and inhibitory neurons, the synaptic density, the density of gap junctions or the ratio between neurons and glia cells.

Although the existence of oscillations did not depend on network architecture, the oscillation frequency differed between clusters grown on PDL and on CNT islands (Figure 4e). This discrepancy may be the outcome of morphology differences dictated by the support substrate. While CNT islands serve as three dimensional highly entangled substrate with which cell mechanically interact [41], PDL islands are flat. Another possible explanation may be related to the increased excitability reported for neurons grown on CNTs surfaces [42]. Further investigation is required to resolve this issue.

Our approach for studying network-level activity focuses on artificially constructed isolated micro-circuits. Alternative approaches can be used to study small scale isolated circuits. Both vertebrates and invertebrates have central pattern generators (CPGs), these are micro-circuits which produce oscillations in absence of any sensory inputs [43]. Indeed, such circuits served as a valuable small-scale model for examining network level interactions, and for unraveling the underlying mechanisms of synchronous activity in large networks [43], [44]. However, there are marked differences between the two approaches. Although CPGs can be considered as analogous to cortical circuits [45], there are some differences. Most importantly, CPGs are developmentally hard-wired to perform a pre-designed task, while cortical circuits are highly plastic and are constantly reshaped by incoming stimuli. In addition, our engineering approach is aimed not only to create a simplified small scale neuronal circuit but rather to design its topology in order to understand how this topology relates to activity. For example, our observation of spontaneous oscillations in isolated clusters with frequencies closely related to those observed in vivo, provide a strong incentive to explore how several connected clusters function. Indeed, our approach allows us to systematically address higher hierarchical levels by examining the activity of two coupled clusters or networks of several connected clusters. Such experiments are currently underway.

Finally, based on the data presented here, it is evident that engineered neuronal networks are a powerful platform to systematically approach questions related to the dynamics of neuronal assemblies. Unlike networks in vivo, in which multiple activation pathways are impinging on any recorded region, isolated networks can be studied in a controlled isolated environment. The high susceptibility to manipulations obtained when working in vitro allows the construction of networks of various sizes, thus enabling the study of scaling properties in networks. Furthermore, engineered networks are highly suited for comparison with modeling results as they allow testing predictions in simplified and pre-designed scenarios.

## Materials and Methods

### Cell culturing

The entire culture preparation process was described in detail in a previous publication [28]. The procedure was done in accordance with the NIH standards for care and use of laboratory animals and was approved by the Tel-Aviv University Animal Care and Use Committee (permit number - M08064). Briefly, entire cortices of (E18) Sprague Dawley rat embryos were removed, chemically digested and mechanically dissociated by trituration. Dissociated cells were suspended in growth medium and plated onto the patterned substrates at a density of 700 cells/mm^2^. To promote the long-term survivability of the cells on the isolated islands it was crucial to use a “feeder” colony of cells [46]. In order to do this, a PDL coated (Sigma, Cat. No. p7889) thin disk of polydimethylsiloxane (PDMS) was placed around the patterned area. The surrounding feeder culture on the disk covered approximately 75% of the total chip area and did not directly contact the patterned culture. The mitotic inhibitor, FuDr (80 µM FuDr, Sigma, Cat. No. F0503 and 240 µM Uridine, Sigma, Cat. No. U3303) was added once after four days in culture. The cultures were maintained at 37°C with 5% CO2 and 95% humidity. The growth medium was partially replaced every 3–4 days.

### Immunostaining

The entire immunostaining process was described in detail in a previous publication [28]. Briefly, neurons, glial cells and neuronal synapses were fixed and immunostained with primary anti-bodies for MAP2 (Abcam, AB2935-ab), GFAP (Biotest, Cat. No. MAB3402) and Synapsin (Biotest, Cat. No. AB1543). These were conjugated with secondary anti-bodies with the following markers: Cy3 (Chemicon, AP194) for MAP2, Alexa fluor 488 (molecular probes, Cat. No. A-11029) for GFAP, and Alexa fluor 546 (molecular probes, Cat. No. A-11035) for Synapsin. For fixed cells, nuclei were stained with 0.01 µg/ml DAPI (4′,6-Diamidino-2-phenylindole dihydrochloride) (Sigma, D9542) for 5 min. For live cells, nuclei were stained with 4 µg/ml bisbenzimide Hoechst 33342 (Sigma, B2261) for 5 min. Finally, samples were mounted using a mounting medium (Sigma, Cat. No. G0918) and covered with a cover slip. Culture images were obtained using both Olympus BX51WI and Zeiss LSM 510 META NLO microscopes.

### Electrophysiological recording

Extra-cellular recording were conducted utilizing low noise pre-amplifiers board (MEA1060-BC amplifier, gain ×1,100 with a band-pass filter of 10 Hz to 3 kHz, by Multi Channel Systems, MCS, Reutlingen, Germany). The signals collected from the microelectrodes were sampled at a 10 kHz sampling rate and stored on a personal computer equipped with a 60 channel, 12-bits data acquisition board (MC_Card, MCS) and a MC_Rack data acquisition software (MCS). An additional 200 Hz high pass filter was applied to the data stream on software. Recordings were performed between 16 to 25 days in vitro. The statistical activity parameters and power spectrum for every cluster were extracted from continuous electrical activity recordings of at least 8 hours. Some of the clusters did not exhibit detectable electrical activity. Since this may be the outcome of damaged electrodes or bad coupling between the electrode and the clusters, these electrodes were removed from the analysis. In total, out of the silent electrodes only 8.5% had clusters with areas larger than 5000 µm^2^ and 70.2% had areas smaller than 1000 µm^2^. However, measuring the cluster size or validating the existence of cells in very small clusters (<1000 µm^2^) could not be properly determined using bright field imaging. Generally, very small clusters are often silent and large clusters are rarely silent.

### Power spectrum analysis

The power spectrum of every cluster was calculated from its CAI activity traces (in 2 ms bins). First, consecutive NBs were extracted (see details in Text S4 and Figure S5). The power spectral density was then estimated using a periodogram [47] for every NB on windows of 2048 ms (NBs with duration less than 2048 ms, were zero padded). Finally, the power spectral density function was averaged over all NBs and then smoothed by convoluting it with a normalized constant function (2.44 Hz wide).

### Patterning isolated network using CNT-MEA

The entire fabrication process was described in detail in a previous publication [48]. Briefly, underlying TiN lines are used as conducting tracks. These lines are passivated with sputtered Si_3_N_4_ which is later removed at the regions of the active electrode using a reactive ion etch step. A thin nickel layer is e-beam evaporated at the openings. The process is concluded with a CNT thermal chemical vapor (CVD) deposition growth procedure utilizing the nickel as a catalyst material. To perform electrical recordings from cultured networks using CNT-MEA chips, clean silicon chips were bonded to printed circuit board (PCB) supports and were adjusted with quartz tubes to contain the biological medium.

### Patterning isolated network using PDL

The process of PDL patterning was detailed in a previous publication [26]. Briefly, PDL islands on top of MEAs were prepared with a soft lithography process using polydimethylsiloxane (PDMS) stencils. An SU8-2075 (Micro Chem) mold with approximately 120 *µ*m thickness is patterned on a silicon wafer. The pattern is identical to the negative pattern of the electrode array. The stencil is prepared by spin coating the wafer with PDMS. After detaching the PDMS substrate from the mold, the stencil is placed on commercial MEAs (MCS) and the stencil's pattern is aligned with the electrode locations. The PDL solution is dripped onto the PDMS stencil and the PDL is dried on a hot plate at 37°C. The PDMS stencil is removed before cell plating.

## Supporting Information

Figure S1 The spatial arrangement of synapses and glia cells in a cluster. (a), (b) and (c) are plane cross-sections perpendicular to the Z,Y and X axes, respectively, of a cluster grown on a CNT electrode (Diameter = 80Î¼m) in which neuronal synapses appear in red (stained with synapsin) and glia cells appear in green (stained with GFAP). The Z, Y and X cross-section planes are represented by the blue, green and red lines, respectively. Clusters are typified by an underlying glia cell layer and mostly overlaying neurons with clearly visible synaptic development.
(0.88 MB TIF)

Figure S2 Correspondence between cluster size (S) and cell number (N). (a) A bright field image of a cluster on a CNT electrode. The cluster area is manually marked (blue) and its area calculated. (b)–(f) A series of fluorescent images of the cluster in (a) taken in consecutive focal planes (from top to bottom) in which live cell nuclei were stained with Hoechst 33342. In each plane the counted cells are marked (blue full circles). (g) Three dimensional reconstruction of a confocal microscope image of four clusters on CNT electrodes. The geometrical shape of the clusters resembles a spherical dome. Electrode diameter is 80 µm and cluster heights are 41 µm (left up), 31 µm (right up), 27 µm (left down) and 43 µm (right down). (h) The number of cell nuclei (N) as a function of the cluster area (S) for several clusters (dots). The blue dot corresponds to the cluster in (a) – (f). The data points were linearly fitted with N = 0.0079*S-1.9 (solid line). The dashed lines represent one standard deviation of the number of cells for all clusters around the fitted curve.
(9.51 MB TIF)

Figure S3 CAI validation. (a) Binary raster plot of spiking activity in a uniform network of cortical neurons during a NB. This activity was recorded by 41 electrodes and was used as a model for the activity in a single cluster. First, the binary spike time series of every single neuron (b) is taken and convoluted with a typical extracellular spike waveform (c) from the same recording to form the convoluted waveform (d). (b),(c) and (d) show the example of neuron number ten (marked by the gray line in (a)). The sum of all convoluted waveforms of all neurons in addition to normally distributed noise is used as a model of the waveform of a cluster during an NB (e). The firing rate (FR) of the cluster during the NB is shown in (f) and the cluster activity intensity (CAI) is estimated according to equation S1 and presented in (g) by a color code. (h) The correspondence between FR and CAI is linear for firing rates of up to 4000 spikes/second. At higher firing frequencies this relation increases monotonically but not linearly. CAI and FR were calculated in time windows of 10 ms.
(2.98 MB TIF)

Figure S4 Cluster activity intensity (CAI) measure. (a) Voltage waveform of a neuronal cluster recorded from a CNT electrode. (b) The unbiased kurtosis of the waveform in (a) calculated for consecutive 20 ms time windows. Windows with kurtosis values below a threshold of three (red line) correspond to windows with noise signals and are chosen for noise level estimation (blue dots). (c) CAI of the waveform in (a) calculated using equation (S1) in 10 ms windows. Negative values (below the red line) correspond to noise segments and their value is set to zero. (d) CAI presented using color coding (same color code as in Figure S3).
(2.22 MB TIF)

Figure S5 NB occurrence and width detection. (a) Example of a CAI (2 ms bins) time series of a NB. (b) The number of CAI active bins (corresponding to non-zero values) in consecutive widows of 10 ms of the time series in (a). (c) The time series in (b) after convolution (convolution kernel is a constant function of one with duration of 100 ms). To eliminate single spikes from this time series, a threshold is applied (threshold = 10 counts). (d) The widened (see text) binary time series of NB locations. (e) The CAI of the NB during the time window marked in black in (d). The exact beginning and ending of the NB are taken as the edges (first non-zero value from both sides) of the CAI function (red lines). (f) The peak time of the NB is taken as the maxima (red circle) of the CAI function after convoluting it with a Gaussian kernel (σ = 50 ms, bin = 10 ms).
(0.76 MB TIF)

Figure S6 Effect of Bicuculline on oscillations. (a) and (b) show the average power spectrum before (blue trace) and after (red trace) the addition of 30Î¼M Bicuculline for a typical isolated and coupled cluster, respectively. In both clusters, a clear oscillation peak was observed before the application of Bicuculline and abolished after application. (c) and (d) show 100 CAI traces of consecutive NBs from the cluster in (a) and (b), respectively, before the addition of Bicuculline. Clear oscillations are observed in individual NB traces before the application of Bicuculline. (e) and (f) show the average CAI profile over all NBs in (c) and (d), respectively. (g) and (h) show 100 CAI traces of consecutive NBs from the cluster in (a) and (b), respectively, after the addition of 30Î¼M Bicuculline. (i) and (j) show the average CAI profile over all NBs in (g) and (h), respectively. The color code in all plots is the same as in Figure S3.
(20.95 MB TIF)

Text S1 Synaptic development and spatial organization of cells in isolated clusters
(0.03 MB DOC)

Text S2 Relation between cluster area and number of neurons
(0.03 MB DOC)

Text S3 The cluster activity intensity (CAI) measure
(0.03 MB DOC)

Text S4 Detecting the occurrence and width of network bursts
(0.03 MB DOC)

Text S5 Effect of GABA blockers on oscillations
(0.03 MB DOC)
