# Supplementary figures and images for: Innate Synchronous Oscillations in Freely-Organized Small Neuronal Circuits
Source: PLoS One. 2010 Dec 28;5(12):e14443. doi: 10.1371/journal.pone.0014443 (PMC3010988; doi:10.1371/journal.pone.0014443)

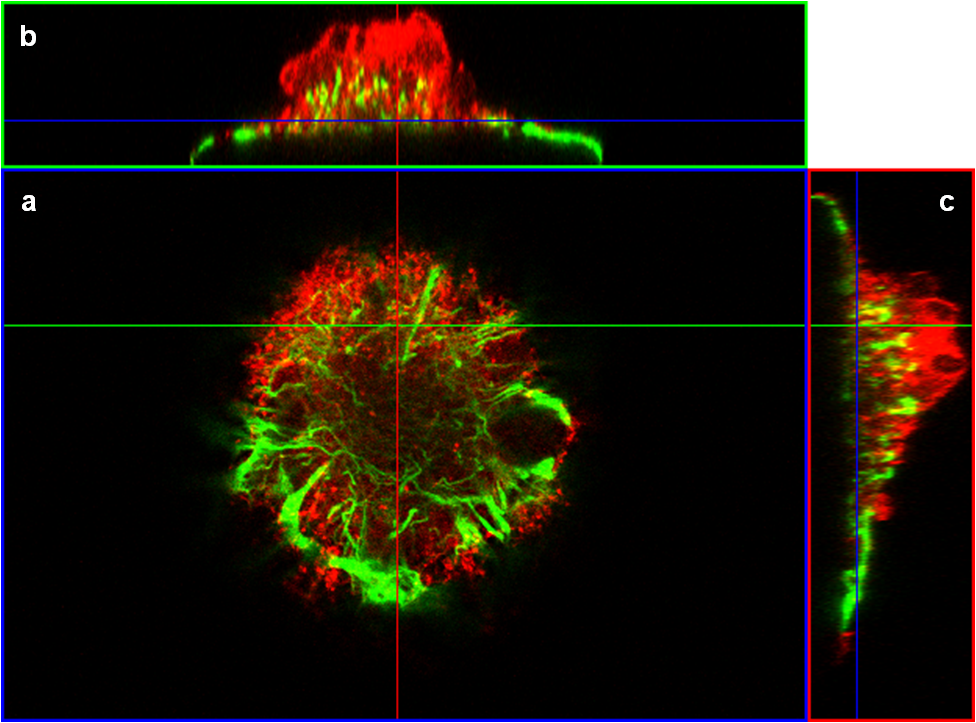

Supplement: Figure S1 — The spatial arrangement of synapses and glia cells in a cluster. (a), (b) and (c) are plane cross-sections perpendicular to the Z,Y and X axes, respectively, of a cluster grown on a CNT electrode (Diameter = 80Î¼m) in which neuronal synapses appear in red (stained with synapsin) and glia cells appear in green (stained with GFAP). The Z, Y and X cross-section planes are represented by the blue, green and red lines, respectively. Clusters are typified by an underlying glia cell layer and mostly overlaying neurons with clearly visible synaptic development. (0.88 MB TIF) [file pone.0014443.s001.tif]

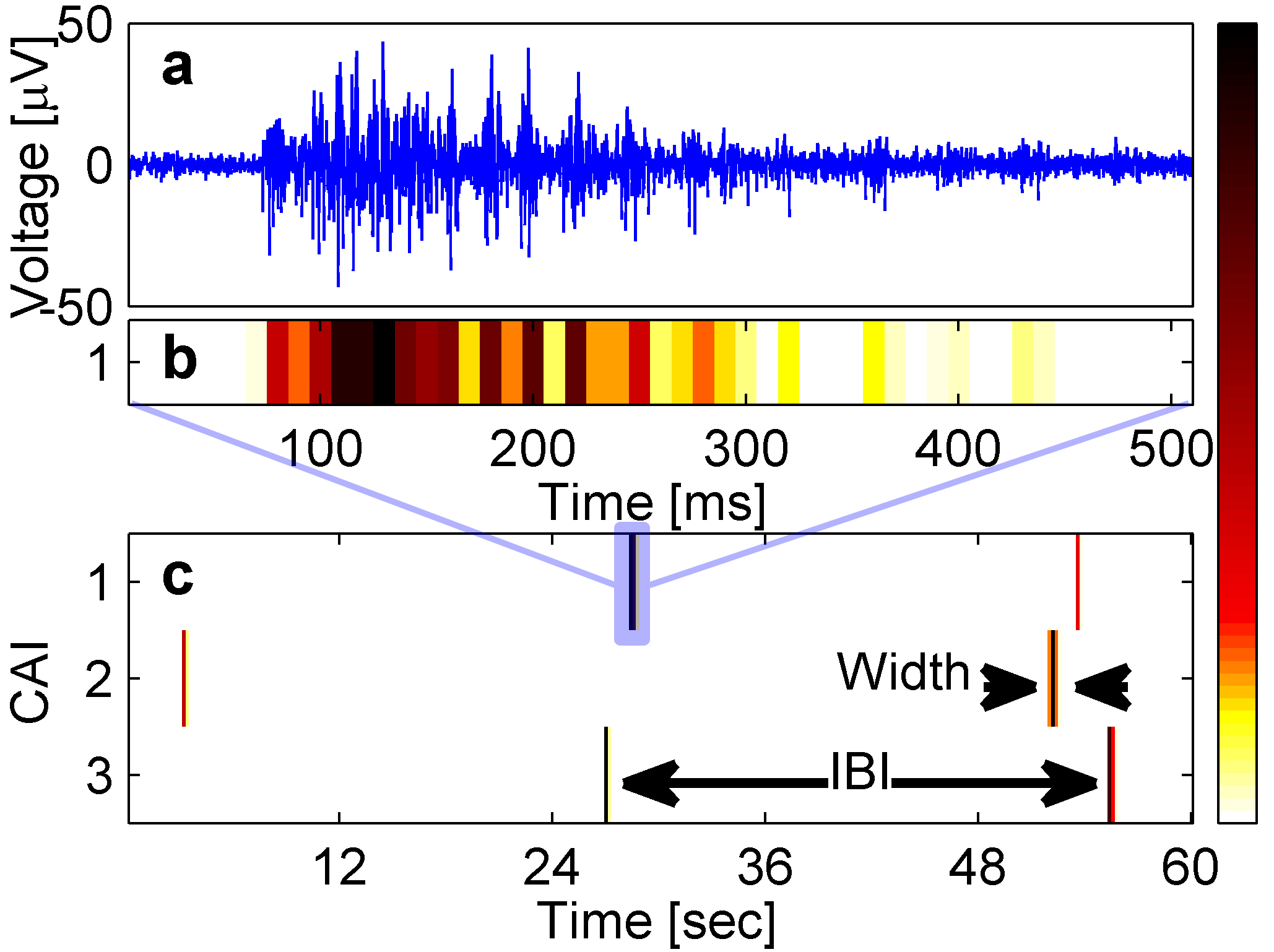

Supplement: Figure S2 — Correspondence between cluster size (S) and cell number (N). (a) A bright field image of a cluster on a CNT electrode. The cluster area is manually marked (blue) and its area calculated. (b)–(f) A series of fluorescent images of the cluster in (a) taken in consecutive focal planes (from top to bottom) in which live cell nuclei were stained with Hoechst 33342. In each plane the counted cells are marked (blue full circles). (g) Three dimensional reconstruction of a confocal microscope image of four clusters on CNT electrodes. The geometrical shape of the clusters resembles a spherical dome. Electrode diameter is 80 µm and cluster heights are 41 µm (left up), 31 µm (right up), 27 µm (left down) and 43 µm (right down). (h) The number of cell nuclei (N) as a function of the cluster area (S) for several clusters (dots). The blue dot corresponds to the cluster in (a) – (f). The data points were linearly fitted with N = 0.0079*S-1.9 (solid line). The dashed lines represent one standard deviation of the number of cells for all clusters around the fitted curve. (9.51 MB TIF) [file pone.0014443.s002.tif]

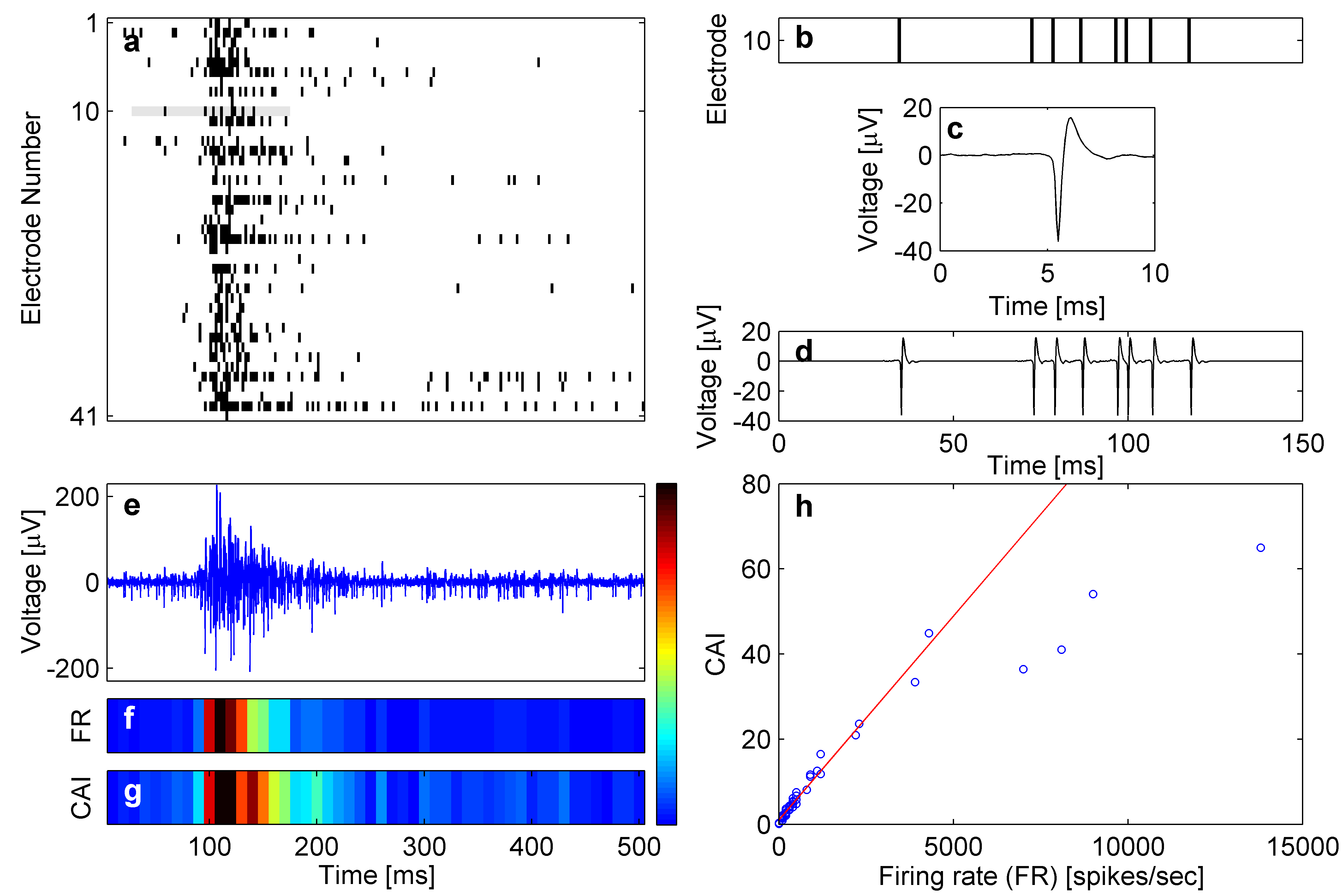

Supplement: Figure S3 — CAI validation. (a) Binary raster plot of spiking activity in a uniform network of cortical neurons during a NB. This activity was recorded by 41 electrodes and was used as a model for the activity in a single cluster. First, the binary spike time series of every single neuron (b) is taken and convoluted with a typical extracellular spike waveform (c) from the same recording to form the convoluted waveform (d). (b),(c) and (d) show the example of neuron number ten (marked by the gray line in (a)). The sum of all convoluted waveforms of all neurons in addition to normally distributed noise is used as a model of the waveform of a cluster during an NB (e). The firing rate (FR) of the cluster during the NB is shown in (f) and the cluster activity intensity (CAI) is estimated according to equation S1 and presented in (g) by a color code. (h) The correspondence between FR and CAI is linear for firing rates of up to 4000 spikes/second. At higher firing frequencies this relation increases monotonically but not linearly. CAI and FR were calculated in time windows of 10 ms. (2.98 MB TIF) [file pone.0014443.s003.tif]

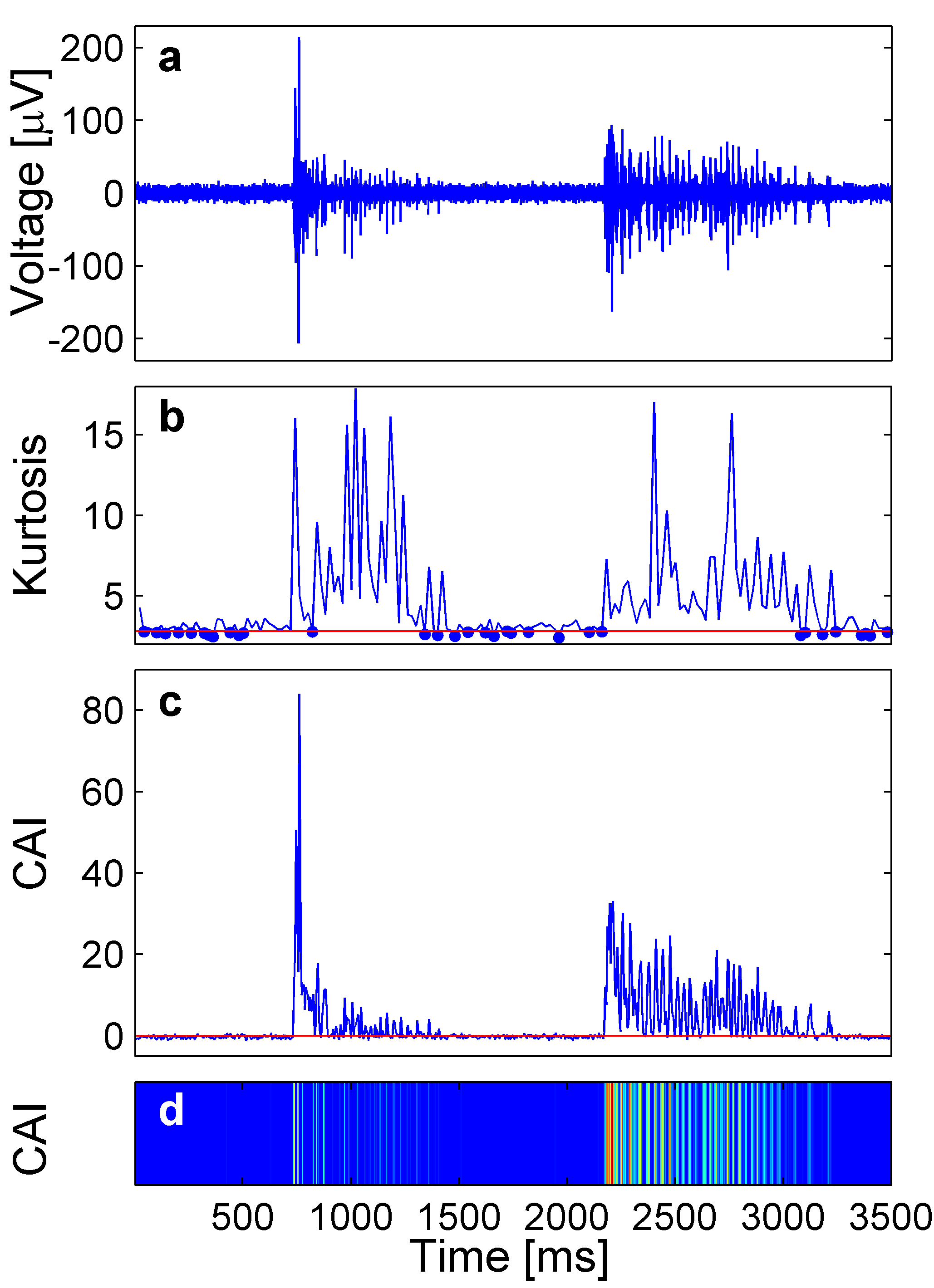

Supplement: Figure S4 — Cluster activity intensity (CAI) measure. (a) Voltage waveform of a neuronal cluster recorded from a CNT electrode. (b) The unbiased kurtosis of the waveform in (a) calculated for consecutive 20 ms time windows. Windows with kurtosis values below a threshold of three (red line) correspond to windows with noise signals and are chosen for noise level estimation (blue dots). (c) CAI of the waveform in (a) calculated using equation (S1) in 10 ms windows. Negative values (below the red line) correspond to noise segments and their value is set to zero. (d) CAI presented using color coding (same color code as in Figure S3). (2.22 MB TIF) [file pone.0014443.s004.tif]

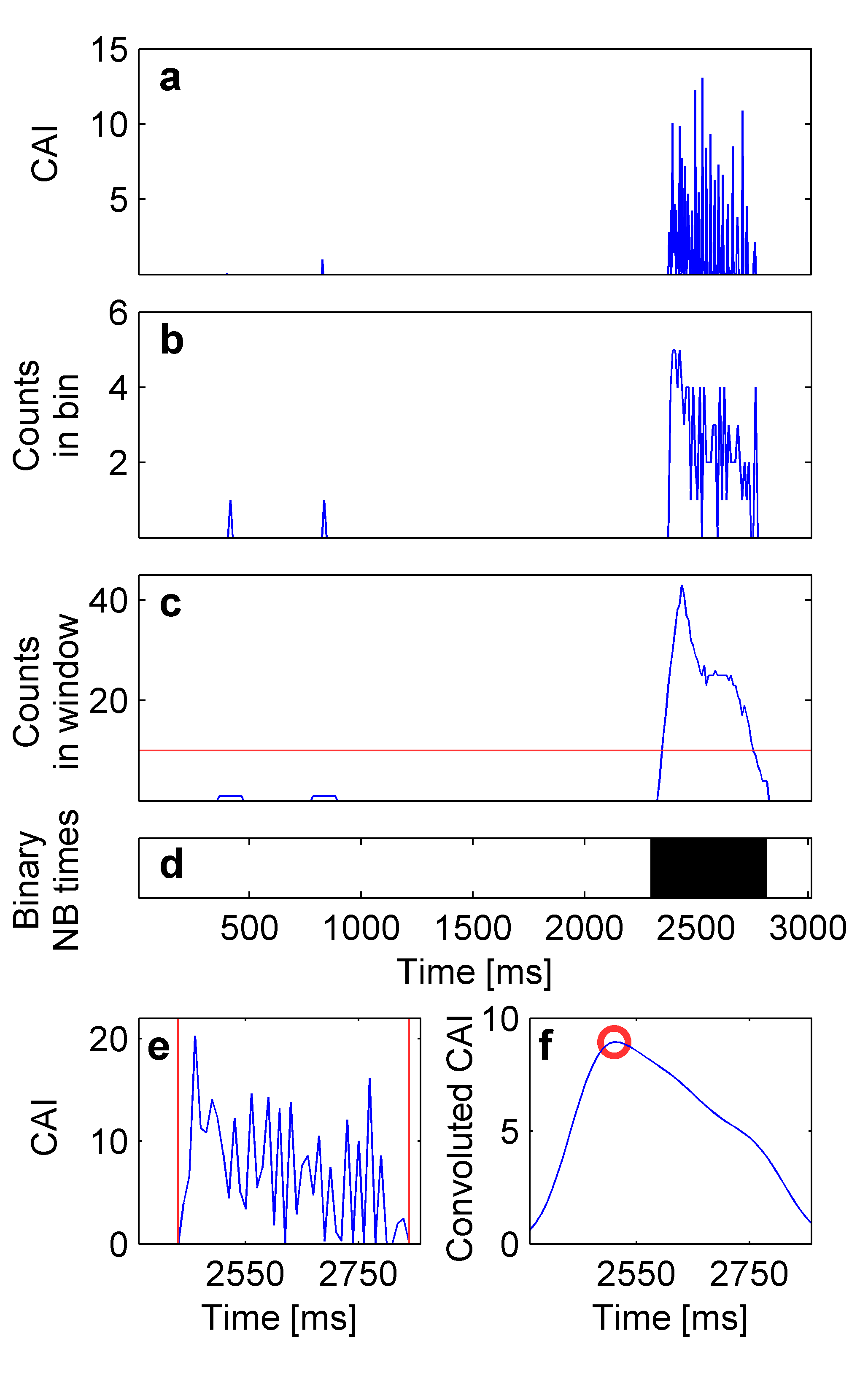

Supplement: Figure S5 — NB occurrence and width detection. (a) Example of a CAI (2 ms bins) time series of a NB. (b) The number of CAI active bins (corresponding to non-zero values) in consecutive widows of 10 ms of the time series in (a). (c) The time series in (b) after convolution (convolution kernel is a constant function of one with duration of 100 ms). To eliminate single spikes from this time series, a threshold is applied (threshold = 10 counts). (d) The widened (see text) binary time series of NB locations. (e) The CAI of the NB during the time window marked in black in (d). The exact beginning and ending of the NB are taken as the edges (first non-zero value from both sides) of the CAI function (red lines). (f) The peak time of the NB is taken as the maxima (red circle) of the CAI function after convoluting it with a Gaussian kernel (σ = 50 ms, bin = 10 ms). (0.76 MB TIF) [file pone.0014443.s005.tif]

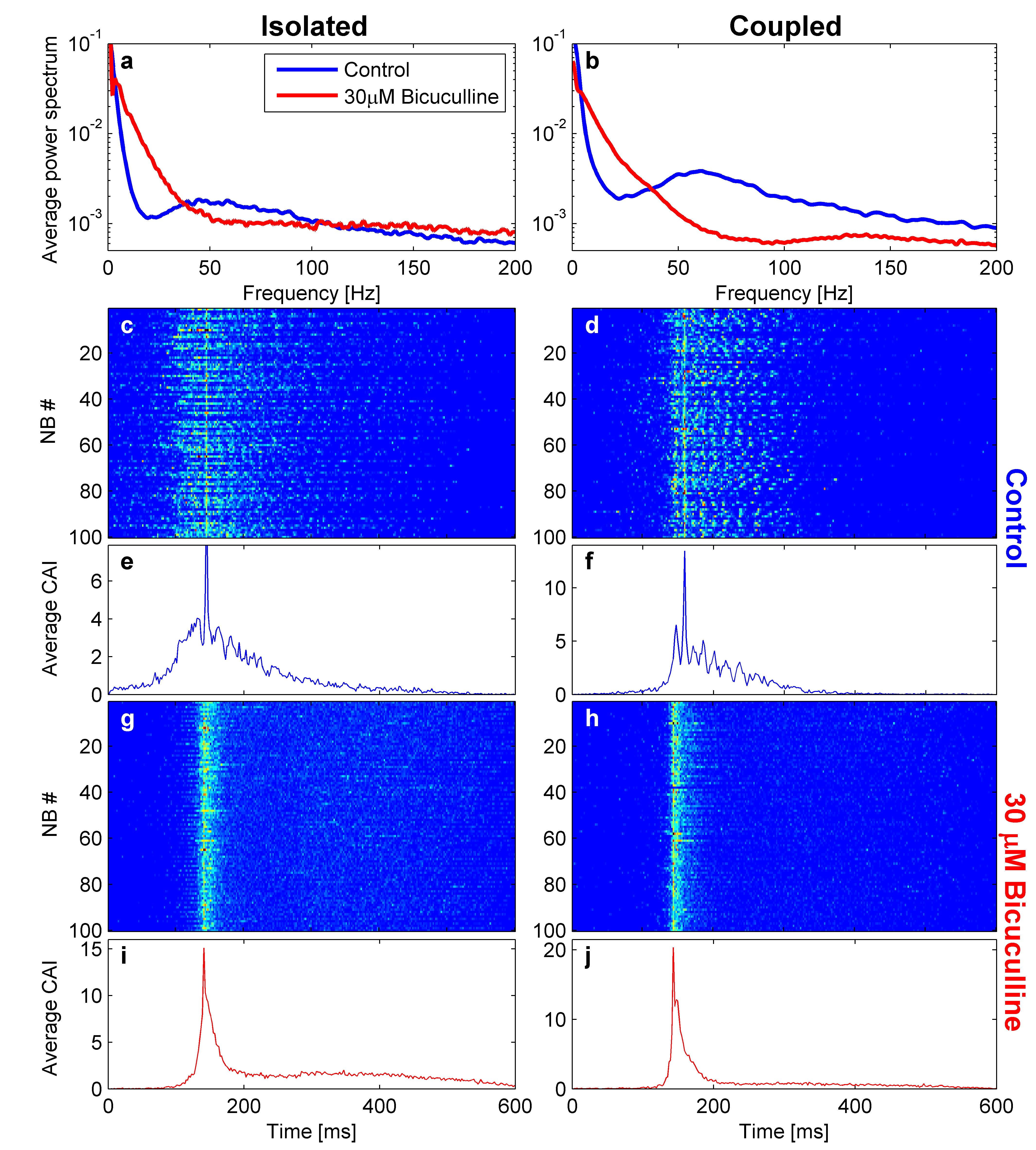

Supplement: Figure S6 — Effect of Bicuculline on oscillations. (a) and (b) show the average power spectrum before (blue trace) and after (red trace) the addition of 30Î¼M Bicuculline for a typical isolated and coupled cluster, respectively. In both clusters, a clear oscillation peak was observed before the application of Bicuculline and abolished after application. (c) and (d) show 100 CAI traces of consecutive NBs from the cluster in (a) and (b), respectively, before the addition of Bicuculline. Clear oscillations are observed in individual NB traces before the application of Bicuculline. (e) and (f) show the average CAI profile over all NBs in (c) and (d), respectively. (g) and (h) show 100 CAI traces of consecutive NBs from the cluster in (a) and (b), respectively, after the addition of 30Î¼M Bicuculline. (i) and (j) show the average CAI profile over all NBs in (g) and (h), respectively. The color code in all plots is the same as in Figure S3. (20.95 MB TIF) [file pone.0014443.s006.tif]
