# Supplementary material for: Innate Synchronous Oscillations in Freely-Organized Small Neuronal Circuits
Source: PLoS One. 2010 Dec 28;5(12):e14443. doi: 10.1371/journal.pone.0014443 (PMC3010988; doi:10.1371/journal.pone.0014443)
Supplement: Text S1 — Synaptic development and spatial organization of cells in isolated clusters (0.03 MB DOC) [file pone.0014443.s007.doc]

**Innate synchronous oscillations in freely-organized small neuronal circuits**

**Supporting information**

**Text S1 - Synaptic development and spatial organization of cells in isolated clusters**

The entangled, three-dimensional arrangement of neurons and glia cells within the clusters was revealed by confocal fluorescent microscopy (Figure S1). Glia cells and synapses were specifically labeled and imaged, revealing that while neurons are arranged in three dimensional conformations, glia cells appear to spread mostly as thin carpets over the CNT surfaces. The extensive formation of synapses provides further support to the notion that clusters of neurons and glia serve as a suitable spatial arrangement for network development.
