# Supplementary material for: Innate Synchronous Oscillations in Freely-Organized Small Neuronal Circuits
Source: PLoS One. 2010 Dec 28;5(12):e14443. doi: 10.1371/journal.pone.0014443 (PMC3010988; doi:10.1371/journal.pone.0014443)
Supplement: Text S2 — Relation between cluster area and number of neurons (0.03 MB DOC) [file pone.0014443.s008.doc]

**Innate synchronous oscillations in freely-organized small neuronal circuits**

**Supporting information**

**Text S2 - Relation between cluster area and number of neurons**

The number of neurons in a cluster was estimated from the cluster area. The area of each cluster was manually measured from its bright field image (Figure S2 a). To relate the measured area with the number of cells in the cluster, the cell nuclei were stained using Hoechst 33342 (see Methods) and counted. Due to the three dimensional arrangement of the cells in the clusters, cell counting was performed in several focal planes to ensure that all cell nuclei were included in the count. Figures S2 (b-f) are a series of images taken in consecutive focal planes (from top to bottom). In each plane counted cells are marked (blue circles). Marked cells are ignored in the cell count of the next (lower) focal plane so that each cell is counted only once.

To determine the geometrical shape of the clusters, three dimensional images of the clusters were reconstructed from images taken using a confocal microscope (Figure S2 g). This examination revealed that the typical shape of a cluster resembles a spherical dome. Figure S2 (h) shows the relation between the measured area (S) and cell count (N) for clusters with areas of 1000-5000μm^2^. The relation was fitted to a linear function using least squares resulting in the following fitted relation: N=0.0079*S-1.9 (solid line in Figure S2 g). Obviously, there is some variability of the data points around the fitted curve, as quantified by the standard deviation of the number of cells of all clusters around the linear approximation (dotted lines in Figure S2 g). This is due to the fact that every cluster is characterized by a unique spatial arrangement.
