# Supplementary material for: Innate Synchronous Oscillations in Freely-Organized Small Neuronal Circuits
Source: PLoS One. 2010 Dec 28;5(12):e14443. doi: 10.1371/journal.pone.0014443 (PMC3010988; doi:10.1371/journal.pone.0014443)
Supplement: Text S3 — The cluster activity intensity (CAI) measure (0.03 MB DOC) [file pone.0014443.s009.doc]

**Innate synchronous oscillations in freely-organized small neuronal circuits**

**Supporting information**

**Text S3 - The cluster activity intensity (CAI) measure**

A single electrode recording from a neuronal cluster does not record single neurons but rather the superimposed activity of many neurons. This is primarily apparent when the firing rate in the cluster is high, as is the case during network bursts (NBs). This high degree of super position of the activity signal from many neurons with variable delays gives rise to highly reduced waveforms that do not allow the discrimination of single spikes. The preferred variable to quantify this activity, on the population level, is the cluster’s average firing rate. Quantifying the firing rate of a group of neurons from a voltage trace of their superimposed spikes is not trivial. Superimposed spikes may cancel out each other due to their bi-phasic nature, resulting in an unfaithful representation of the activity.

To examine how the superposition of spikes is reflected in their voltage trace, we constructed an artificial waveform of a neuronal cluster by superimposing the voltage waveform of single neurons. For this purpose, the binary spike time series’ of single neurons recorded during a NB in a uniform neuronal network (Figure S3 b) was first convoluted with a typical extra cellular spike waveform (Figure S3 c). Superimposing the convoluted waveforms (Figure S3 d) of all neurons and adding normally distributed noise results in an artificial model waveform of a neuronal cluster recorded by a single electrode (Figure S3 e). It is evident that the increase in the cluster’s firing rate in this model (Figure S3 f) corresponds to the increase in the voltage waveform (Figure S3 e), however, it is unclear how such an increase should be quantified. To resolve this issue, we used the sum of the absolute value of the voltage in consecutive time windows as a measure of the cluster’s activity intensity (CAI):

$$CAI=\left\{\begin{matrix} A, A\geq0 \\ 0, A<0 \end{matrix}\right. , A=\frac{\sum_{i=1}^{M} \left|V(i)\right|}{M}-NT (Eq. S1)$$

where V is the waveform voltage, M is the number of samples in each activity intensity bin and NT is the activity intensity noise threshold. The noise threshold is added in order to remove the contribution of noise to the CAI value and is calculated as follows: First, the unbiased kurtosis (Figure S4 b) of the voltage waveform (Figure S4 a) is calculated in time bins of 20ms, in order to estimate the Gaussianity of every bin (normally distributed values have a kurtosis of 3 while spiking waveforms were found to have much higher values). Next, bins with kurtosis values higher than 3 are rejected. The waveforms of the rest of the signals are used to estimate the average absolute value of a noise voltage sample (Figure S4 b - blue dots), which is the noise activity intensity threshold, NT. Once NT is obtained, CAI is calculated using equation S1 (Figure S4 c,d). To test the performance of our defined CAI as a measure of the firing rate in the cluster, we calculate the CAI (Figure S3 g) of an artificially generated cluster waveform (Figure S3 e) with well known exact firing rate (Figure S3 f). We used data recorded by 41 electrodes and treated it as a cluster data (see above). Figure S3 (h) shows that there is a linear correspondence between the CAI measure and the firing rate, up to total cluster firing rates as high as 4000 spikes/sec. For higher spike rates, CAI as a function of the firing rate is still monotonic, however it is no longer linear.

Different electrodes exhibited different signal to noise levels. This may be the outcome of both the electrode fabrication quality and the coupling between the neurons and the electrode. Consequently, electrodes exhibiting low SNR values were rejected and not analyzed. Additionally, due to the same coupling differences, the CAI cannot be used to compare the absolute firing rates between different electrodes. However, it can be used to compare relative changes in firing rates between different electrodes and absolute firing rates between different time points on the same electrode.
