# Supplementary material for: Innate Synchronous Oscillations in Freely-Organized Small Neuronal Circuits
Source: PLoS One. 2010 Dec 28;5(12):e14443. doi: 10.1371/journal.pone.0014443 (PMC3010988; doi:10.1371/journal.pone.0014443)
Supplement: Text S4 — Detecting the occurrence and width of network bursts (0.03 MB DOC) [file pone.0014443.s010.doc]

**Innate synchronous oscillations in freely-organized small neuronal circuits**

**Supporting information**

**Text S4 - Detecting the occurrence and width of network bursts**

To detect the occurrence (the peak activity time) and width of every network bursts (NBs) we did the following. Our first aim was to transform the recorded activity data into a binary time series in which the temporal occurrence NBs is represented by a consecutive series of ones separated by zeros. To do so, we first calculated CAI in bins of 2ms (Figure S5 a). Next, we counted the number of active CAI bins (having non-zero values) within 10ms windows (Figure S5 b). In the resulting time series, windows with higher bin counts were associated with NB occurrences, however, zero values still appeared within NBs, parsing them to several sub-events. To eliminate such values we convoluted this time series with a constant kernel function of value one and length W (W=100 ms). Such a convolution is analogues to applying a floating window which counts the number of bins in a window of length W around each point (Figure S5 c). To eliminate single spikes from the time series of NBs, we applied a threshold of value T (T=10) (Figure S5 c - red line), so that data points with values below T were zeroed. In the Bicuculline experiments (see Text S5), both before and after the application of Bicuculline we used T=30, which yielded a more accurate burst detection. To ensure that activity near the NB edges was included in the NB, a second convolution with the same kernel followed by thresholding with T=1 was performed. Finally, NBs occurring less than 100ms apart (ending of previous to beginning of next) were merged into one NB. This process resulted in a binary time series of NB occurrences with a temporal resolution of W (Figure S5 d). To increase the accuracy of the NB start time, end time, and peak time detection, the CAI function (in 10ms resolution) was extracted during the time windows of the NB occurrences. The beginning and ending of NBs were taken as the first non zero value from left and right, respectively (Figure S5 e – red lines). The peak of the NB is determined by smoothing the CAI (using a convolution with a Gaussian, σ=50ms) and extracting the maxima (Figure S5 f – red circle).
