# Supplementary material for: Innate Synchronous Oscillations in Freely-Organized Small Neuronal Circuits
Source: PLoS One. 2010 Dec 28;5(12):e14443. doi: 10.1371/journal.pone.0014443 (PMC3010988; doi:10.1371/journal.pone.0014443)
Supplement: Text S5 — Effect of GABA blockers on oscillations (0.03 MB DOC) [file pone.0014443.s011.doc]

**Innate synchronous oscillations in freely-organized small neuronal circuits**

**Supporting information**

**Text S5 - Effect of GABA blockers on oscillations**

To verify that the oscillations are not an artifact of our unique preparation, we applied 30μM Bicuculline (GABA receptor antagonist)(Sigma , Cat. No. 14343) to clusters grown on PDL islands. The addition of Bicuculline resulted with a clear removal of the oscillations, both in isolated clusters (Figure S6 a) and in coupled cluster (Figure S6 b). The effect of Bicuculline is also reflected in the activity traces of single bursts (Figure S6 c,d,g,h) and the average NB profiles (Figure S6 e,f,i,j). In total, eight clusters were examined, four isolated and four coupled clusters. Two out of four isolated clusters and four out of four coupled clusters exhibited clear oscillation peaks in their averaged power spectrum. In all clusters, these oscillation peaks were abolished following the application of Bicuculline.
